# The acceptability, effectiveness, and impact of different models of care for pediatric weight management services: protocol for a concurrent mixed-methods study

**DOI:** 10.1186/s12913-018-3222-7

**Published:** 2018-06-07

**Authors:** Jennifer Cohen, Shirley Alexander, Michelle Critekos, Sarah P. Garnett, Alison J. Hayes, Tim Shaw, Kyra A. Sim, Louise A. Baur

**Affiliations:** 1 Weight Management Services, The Children’s Hospital at Westmead, Westmead, NSW Australia; 2 Discipline of Pediatrics, School of Women’s and Children’s Health, UNSW Medicine, UNSW Sydney, Sydney, NSW Australia; 3 Clinical Quality and Safety, Centre for Population Health, NSW Ministry of Health, North Sydney, NSW Australia; 4 Institute of Endocrinology and Diabetes, The Children’s Hospital at Westmead, Westmead, NSW Australia; 5 Faculty of Medicine & health, Sydney School of Public Health, The University of Sydney, Sydney, NSW Australia; 6 Faculty of Health Sciences, The University of Sydney, Sydney, NSW Australia; 7 Boden Institute, Charles Perkins Centre, The University of Sydney, Camperdown, NSW Australia; 8 Sydney Local Health District, Camperdown, NSW Australia; 9 Discipline of Child & Adolescent Health, The University of Sydney, Sydney, NSW Australia

**Keywords:** Pediatric obesity, Treatment, Implementation research, Models of care, Childhood obesity

## Abstract

**Background:**

Pediatric obesity is a serious, but clinically neglected, chronic health problem. Despite the high prevalence, excess weight problems are rarely managed when children attend clinical services. It is recommended that obesity treatment uses a “chronic-care” approach to management, with different types and intensity of treatment dependent upon severity of obesity. There are several new secondary and tertiary weight management services being implemented within New South Wales (NSW), Australia in 2017/2018 with differing models of care. This study will ascertain what factors affect acceptability, reach, and participation, as well as measure the clinical effectiveness of these services.

**Methods:**

This is a acceptability and effectiveness study building upon existing and planned secondary and tertiary level service delivery in several health districts. This study will recruit participants from seven different pediatric weight management services (PWMS) across five Local Health Districts in NSW, Australia. Using a mixed-methods approach we will document a range of process, impact and clinical outcome measures in order to better understand the context and the effectiveness of each PWMS model. The project development and implementation is guided by the Theoretical Domains Framework. Participants will include parents of children less than 18 years of age attending PWMS, clinicians working as part of PWMS and health service managers. Data will be captured using a combination of anthropometric measures, questionnaires, one-on-one semi-structured interviews and focus groups.

**Discussion:**

Results from this study will assess the acceptability and effectiveness of different models of care for pediatric weight management. Such information is required to inform long-term sustainability and scalability of secondary and tertiary care services to the large number of families with children above a healthy weight.

## Background

Pediatric obesity is a serious, but clinically neglected, chronic health problem. In Australia one in four school-aged children and adolescents is affected by overweight [1] or obesity (6% with obesity, a further 2% with severe obesity) [2]. Pediatric obesity is associated with a range of immediate and longer-term health problems [3]. Despite the high prevalence, excess weight problems are infrequently managed when children attend clinical services [4, 5]. Barriers to weight management by health professionals include: difficulty raising the issue, uncertainty about advice to offer and referral pathways, and lack of local service capacity [5, 6].

Untreated, the natural history of obesity in children and adolescents will worsen over time [7]. The American Academy of Pediatrics recommends that obesity treatment uses a “chronic-care” approach to management, with different types and intensity of treatment dependent upon severity of obesity [8]. Family-based lifestyle interventions incorporating behavior modification around eating and activity are effective and considered first-line treatment for pediatric obesity [3, 9, 10], especially in younger children [11]. Although there is a central role for primary care physicians and other health professionals, including nurses and dietitians, in the management of childhood overweight and obesity [12], there is also a need for specialist secondary and tertiary care services for those with more severe obesity [3]. Primary, secondary and tertiary care services can all provide dietary modification, advice about exercise and physical activity [12], medical management of obesity-associated complications, family-based lifestyle changes and behavior modification techniques [3]. Secondary and tertiary services differ from primary care services by providing more intensive services within a multi-disciplinary team environment and for children with more severe obesity.

Australia has a publicly funded healthcare system [13]. The individual states and territories are responsible for managing hospital and community services [14]. Primary care is the first contact with the health system and does not include hospital services. Secondary care services are provided by specialist care providers and are a direct link between primary and tertiary services. Within New South Wales the state is divided into eight metropolitan local health districts (LHDs) and seven rural LHDs. In addition, there are two specialist networks, including the Sydney Children’s Hospitals Network (SCHN).

There are limited pediatric obesity services in Australia, including in the state of NSW. Prior to 2017, the number of services available in the secondary and tertiary setting had not changed in the previous ten years [15], despite the number of children with severe obesity significantly increasing. The few existing services are poorly linked, with no well-established models of care or healthcare pathways across primary, secondary or tertiary care networks. Adequate treatment strategies for childhood obesity will require changes in healthcare delivery systems [16].

Several LHD’s within NSW are implementing new services to deliver integrated weight management programs for children and adolescents. The new and existing services vary greatly in their models of care, depending upon local resourcing, clinical needs, staff expertise and patient characteristics. We have a unique opportunity to research the implementation and integration of these pediatric weight management services (PWMS) alongside established services to determine the acceptability, clinical effectiveness and impact of the services. There is limited research assessing the effectiveness of interventions in clinical practice for children with obesity [17] . Inadequate documentation and poor retention rates of patients are factors shown to prevent the assessment of “real-world” models of care for PWMS in international settings [17]. No such research has been undertaken previously in Australia.

### Primary objective

- To assess the acceptability, reach, participation rates and retention rates of different models of care for management of childhood obesity at the secondary and tertiary level care.

### Secondary objectives

- To compare the clinical effectiveness of the different models of care for the management of childhood obesity
- To assess the impact of the different pediatric weight management models of care on weight-related behaviors (eating, physical activity and sedentary behaviors).
- To determine the costs and cost-effectiveness of the different models of care from a health provider and consumer perspective.

## Methods/design

We have designed a multi-perspective, concurrent, mixed-methods study testing the appropriateness, acceptability, clinical effectiveness, and impact of secondary and tertiary level PWMS. It will harness the opportunity provided by the development of new PWMS in the state of NSW in 2017/2018 (a “natural experiment”). Qualitative and quantitative approaches will be used to gather evidence and assess the existing and new models of care. We will document a range of process, impact and clinical outcome measures in order to better understand the context and the effectiveness of each child and family weight management service model. Health economic evaluation of different models of care will also be undertaken.

The research will be guided by the Theoretical Domains Framework [18]. A co-design framework and Linkage-Exchange Model will be applied throughout this project [19]. This will ensure that patients, families, clinicians and managers are engaged in the project at all stages of development and that a systematic and theoretically underpinned model is used to disseminate findings into practice and policy [19]. Linkage and exchange strategies involve initiatives that seek to i) promote research use in decision contexts, and ii) encourage research that generates evidence that is of use to decision-makers. Interpersonal connections (interactions) between clinicians, managers, patients/families are vital in this process.

### Study setting

The study will be undertaken at six secondary level care clinics and one tertiary clinic for child weight management. These clinics are limited to children less than 18 years of age and their families. Each clinic uses a different model of care with a combination of face-to-face and group education sessions within a multi-disciplinary team.

### Study activities

Parts 1, 2 and 3 of the study will address the following objectives:

Primary Objective: To assess the acceptability, reach, participation rates and retention rates of different models of care for management of childhood obesity at the secondary and tertiary level care.

### Part 1: Assessment of parent/ guardian views of the acceptability of the secondary weight management service

In part 1 of the study we will utilize a cross-sectional assessment of parent acceptability, satisfaction with the PWMSand burden on the family attending the services determined by a questionnaires and one-on-one interviews. Participants will be recruited at completion of the weight management program or at three months into the program for those attending programs with a longer follow-up.

#### Participants

Parents/carers of a child who has been attending secondary weight management service and are fluent in English will be included in this study.

#### Quantitative data

Patient satisfaction will be measured using the Short Assessment of Patient Satisfaction Measure [20]. The perception families have of the empathy of their clinician will be measured using the Consultation & Relational Empathy Measure (CARE) [21] which has good reliability, internal consistency and validity [22]. Parental expectation on treatment outcomes will be assessed using a modified version of the Treatment Expectations and Goals Survey Tool [23]. Additional questions, developed by the multi-disciplinary research and clinical team, on time taken to attend appointments, length of appointment and accessibility of the service will be asked using a 5-point Likert scale from “strongly disagree” to “strongly agree”. The multi-disciplinary team included researchers, pediatricians, dietitians, nurses, psychologists and project officers. The questionnaire should take no longer than 20 min to complete.

#### Qualitative data

Parents/ carers who complete the questionnaire will be able to opt-in for one-on-one telephone interviews. Semi-structured telephone interviews will capture parent views on: 1) The acceptability of the service design and delivery (i.e. service location, accessibility, cultural appropriateness, language used, context and acceptability of intervention); 2) perceptions of the services’ strengths and weaknesses; 3) barriers and enablers to attendance at the service and barriers and enablers to behavior change; 4) views on education materials and resources provided by the weight management clinic; and 5) recommendations for improvement. An interview discussion guide has been developed by the multi-disciplinary team based on previous work assessing parental views of weight management services [24, 25].

#### Recruitment

Potential participants who meet the inclusion criteria will be identified by the primary investigators at each site. The project officer will approach the potential participants during their clinic visits and provide the patient information sheet and consent form. If the participant has provided written consent to take part in the study, the project officer will administer the parent questionnaire. At the end of the questionnaire, participants will have the option to consent for the one-on-one interviews.

It is anticipated that a total of 140 participants will complete the parent questionnaire. As this is a non-randomised acceptability study, a formal sample size calculation could not be performed. The number of participants was a pragmatic decision based on the number of potential participants from each clinic during the study timeframe. This pragmatic approach to sample size calculations has been used previously [26] and is within the acceptable range for sample sizes for quantitative analysis in mixed-methods approaches [27].

Consenting participants will then be contacted by phone by the project officer to invite them to participate in a one-on-one interview. Maximum variation sampling will be used to ensure a range of ages, severity of obesity and sex of the children attending the weight management clinics and a range of clinicians to ensure we capture a broad spectrum of perspectives and experiences [28]. The interviews will continue until data saturation is reached with no new themes emerging. It is estimated that 50 families will take part in the one-on-one interviews. These numbers are based on previous studies assessing parent views [29–31].

### Part 2: Assessment of clinician views of secondary services for children who are above their healthy weight range

In part 2 of the study clinician views of the PWMS they work in, the service they provide and their experience working in that service will be assessed by questionnaire and focus groups. The following information will be collected: 1) staffing mix; 2) expertize; 3) training; 4) time commitments; 5) service model; 6) level of multi-disciplinary team work; 7) internal and external department links; 8) costs and funding sources.

#### Participants

Participants will be clinicians who are involved with the care of patients in a secondary or tertiary PWMS. Clinicians will include pediatricians, dietitians, nurses, psychologists and physiotherapists. Clinicians will be given a patient information sheet and written consent will be obtained before taking part on the study.

##### Quantitative data

A questionnaire, developed by the multi-disciplinary research team and clinical team, will be used to capture clinician views about their PWMS. The questionnaire is based on a previously published survey [19]and includes questions on respondent demographics and experience in pediatric weight management, clinic levels of service and demands, weight management service model, approaches to weight management. The questionnaire will take no longer than 20 min to complete.

##### Qualitative data

Focus groups will further explore information gained from the survey and aim to capture: 1) perceptions of service strengths and weakness; 2) barriers and enablers to a successful PWMS; 3) previous experience with non-sustainable services; 4) training needs of clinicians working in pediatric weight management; 5) suggested resources; 5) recommendations for improvement. The semi-structured interview guide for the focus groups was developed by a multi-disciplinary group of researchers and clinicians.

#### Recruitment

We will purposively sample clinicians to ensure we capture a broad and multidisciplinary range of perspectives [28]. The project officer will approach the clinicians via email or in person during clinic visits. All participants will be advised (verbally and in writing) that participation is voluntary and they are able to withdraw from the study at any point without reason or consequence. The potential participants will be provided with the study information sheet and consent form. If the participant has consented to take part in the study, the researcher will administer the questionnaire in person or will send a link to the questionnaire. At the end of the questionnaire, participants will have the option of consenting to participate in the focus groups. Clinicians will then be contacted by phone or email by the project officer to invite them to attend a focus group. It is estimated that 15–20 clinicians will take part in a focus group. This number is feasible based on previous studies of clinician views of PWMS [32, 33].

### Part 3: Health service manager views of secondary weight management services for children who are above their healthy weight range.

Part 3 of the study is a qualitative investigation of the views of health service managers of the implementation of PWMS.

#### Participants

Health Service Managers who manage health services that have a secondary or tertiary PWMS. Appropriate Health Service Managers will be recruited via email and will be given a patient information sheet and consent form for written consent.

#### Qualitative data

One-on-one interviews, using semi-structured interviews will be used to assess the views of health service managers. 1) LHD views of the PWMS; 2) future plans for the service; 3) funding considerations; 4) linkage and integration with related services; 5) perceptions of strengths and weaknesses of the pediatric weight management service; 6) barriers and enablers; 7) recommended resources; 8) recommendations for improvement for future PWMS.

#### Recruitment

Health Service Managers who meet the inclusion criteria will be identified by the primary investigators at each site. The project officer will approach the Health Service Managers via email. The researcher will then book in a time to conduct the one-on-one interview either in person or via the telephone at a time that suits the participant.

### Part 1, 2 & 3 qualitative data analysis

Responses from the focus groups/ one-on-one interviews will be recorded on a high-quality recorder and the interviews will be transcribed verbatim by an independent researcher. Two investigators will listen to the interviews and brief synopses of the emergent themes raised in each focus group/ interview will be prepared. Each transcript will then be coded by one of the investigators experienced in qualitative research methods using the qualitative data analysis software package, NVIVO 11 (QSR International PTY LTD). We will conduct content analysis of the qualitative data and the conceptual frameworks of Miles and Huberman (1994) [34] will be used to guide data analysis.

A random sample of 20% of the transcripts will be independently coded and cross checked for inter-rater reliability. Once coding is complete, the coders will meet to review the coding and address any coding disagreements. If the coding pair cannot agree on how to code a particular passage, the issue will be referred to the project manager. If the larger group cannot resolve the problem, the passage will be omitted from the analysis. This multilevel consensus coding method has been applied in multiple settings and meets accepted standards of reliability and validity in qualitative research [34]. The final coding will be analyzed and key response categories will be identified and enumerated [34]. The reporting of the results of the qualitative data collection and analysis will be guided by the COREQ criteria [35].

### Part 4: Efficacy of Services for Children Attending Secondary Weight Management Services

Part 4 is an audit of clinic notes and medical records of data collected routinely as part of standard clinical care for pediatric weight management.

Part 4 will address the following objectives:

Secondary objective: To compare the clinical effectiveness of the different models of care for the management of childhood obesity.

Secondary objectives: To assess the impact of the different pediatric weight management models of care on weight-related behaviors (eating, physical activity and sedentary behaviors).

#### Participants

All children attending the weight management clinics will be included in part 4 of the study.

#### Data collection

The Healthy Lifestyle Questionnaire was developed for the collection of routine clinical data. This questionnaire includes selected validated questions from the NSW Population Health Survey [36] and the NSW Schools Physical Activity and Nutrition Survey [37]. The questions include data on participants’ demographics, screen time, moderate to vigorous physical activity per day, water consumption, milk consumption, sugary drink consumption, breakfast consumption, consumption of some specific snacks, fruit and vegetable consumption and sleep time. Clinicians complete a section on the patient’s height, weight and co-morbidities as part of routine clinical care. These data will be collected at baseline and at three and six months.

#### Data analysis

Data analysis will be performed using appropriate statistical software. Data will be presented descriptively including frequency distributions. Differences in parental and clinician satisfaction between models of care will be examined by comparison of means (continuous data) or Chi-squared (categorical data). Effectiveness of each model of care will be determine by change in BMI z-scores, expressed as a continuous variable and categorical variable (change ≤5% or > 5%), from baseline to 3 and 6 months. Weight changes in children with obesity are associated with change in metabolic outcomes [38]. Predictors of effectiveness, including models of care, eating behaviors and physical activity, will be examined by modelling.

### Part 5: Economic evaluation of each model of Care for Pediatric Weight management services

Part 5 will address the following objective:

Secondary objective: Determine the costs and cost-effectiveness of the different models of care from a health provider and consumer perspective.

We will determine the costs and cost-effectiveness of weight management services from the perspective of the health care provider and patients and their families. We will consider not only the cost of setting up services for child weight management in each of the participating LHDs, but also any out-of-pocket costs borne by patients and their families. Health system costs will include fixed and variable costs including clinic set up, staffing costs based on staff full time equivalents, consumables and materials purchased for the running of the clinics. We will use standard techniques to consider overhead costs based on square meters of space occupied within existing hospital or clinic premises. These costs will primarily be based on financial records from LHDs. Additionally, there will be ongoing healthcare costs (e.g. general practitioner, specialist and tertiary services) as each child moves through the care pathway – these will include health system and out-of-pocket costs to patients.

#### Participants

As part of the questionnaire developed for Part 1 of the study (consented) parents/carers will be asked about their time inputs as they navigate the care pathway e.g. time to attend consultations and travel time, type of services used, including specialist services and use of organised programs.

#### Quantitative

Participant costs will include any out-of-pocket costs for referrals, and treatment plus productivity costs relating to missed work. Participant and family costs will be important in assessing the equity and reach of the programs and may impact on the acceptability of different models of care. In addition, we will determine health system costs using standard costs for consults. Hence, for each LHD we will be able to estimate the mean cost per child using the service and total set-up costs required for expansion into other LHDs.

#### Data analysis

The primary outcome for the economic evaluation will be incremental cost per unit BMI z-score avoided in the new model of care compared with the existing model of care. Incremental cost-effectiveness ratios (ICERs) will be determined for each model of care, in each LHD.

## Discussion

This is a unique time in NSW, Australia when several health services are currently working collaboratively with the aim of implementing secondary level care of child weight management in an integrated fashion. However, the models of care to be implemented differ based on the varying level staffing and intensity of the intervention. There have been no studies examining the cost, impact, appropriateness and acceptability of various integrated child and family-focused clinical models of care in secondary and tertiary PWMS within Australia. The current or planned clinical services are all based on the same broad principles of clinical management of pediatric obesity. However, it is important to test the clinical effectiveness of such models of care as the intensity, cost and cost-effectiveness is likely to differ based on the model used.

Results from the study will enable recommendations for future service planning to optimise scalability and sustainability of integrated, responsive and effective care of pediatric patients with overweight or obesity. This study will help to generate an evidence base for secondary and tertiary level weight management models of care in participating LHDs.

The findings from this project will feed directly in to the NSW Premier’s Priority work on “Tackling Childhood Obesity”. The project will improve the design and delivery of local services for children and their families, by enhancing the accessibility, acceptability, reach and impact of services for children and families from diverse regions of NSW. Results from this study will assist in examining ways to better integrate secondary and tertiary pediatric weight management with primary care management and existing community-based programs. As a result, health service managers and senior clinicians will be better informed about the types of models of care and health care pathways that may work best in their region or health service. Such information is required to inform long-term sustainability and scalability of secondary and tertiary care services to the large number of families with children above a healthy weight. Further, children and adolescents affected by obesity will have improved access to secondary level treatment services, which are currently very limited.

## Abbreviations

BMI: Body Mass Index
LHD: Local Health District
NSW: New South Wales
PWMS: pediatric weight management service(s)
